# Long-term biodiversity survey data of Mianhua and Huaping Islets Wildlife Refuge, Taiwan

**DOI:** 10.3897/BDJ.14.e209979

**Published:** 2026-10-14

**Authors:** Jui-Wen Chang, Hsin-Han Tsai, Melissa Jean-Yi Liu, Chung-Hang Hung, Yen-Ting Huang

**Affiliations:** 1 Ceta explorers Co., Ltd., New Taipei City, Taiwan Ceta explorers Co., Ltd. New Taipei City Taiwan; 2 Asia Regional Support Team, Global Biodiversity Information Facility (GBIF), Taipei, Taiwan Asia Regional Support Team, Global Biodiversity Information Facility (GBIF) Taipei Taiwan; 3 Taiwan Biodiversity Information Facility (TaiBIF), Taipei, Taiwan Taiwan Biodiversity Information Facility (TaiBIF) Taipei Taiwan; 4 Hydrotech Research Institute, National Taiwan University, Taipei City, Taiwan Hydrotech Research Institute, National Taiwan University Taipei City Taiwan

**Keywords:** ecological monitoring, occurrence data, protected area, streaked shearwater, volcanic islet

## Abstract

**Background:**

Mianhua and Huaping Islets constitute the legally designated Mianhua and Huaping Islets Wildlife Refuge (hereafter, the Wildlife Refuge), whereas nearby Pengjia Islet lies outside the refuge boundary, but was included in some survey years owing to its geographic proximity or specific survey objectives. In 1996, the Wildlife Refuge was designated by the Keelung City Government (the local competent authority) under the jurisdiction of the Forestry Bureau, Council of Agriculture (the central competent authority, now reorganised as the Forestry and Nature Conservation Agency, Ministry of Agriculture). Since its designation, access to the Wildlife Refuge has required prior permission and has been restricted to research and educational purposes. In 2018, the central competent authority was transferred to the Ocean Affairs Council and its affiliated agencies. Ecological surveys, associated with the Wildlife Refuge, have been conducted from 1994 through 2025 by various implementing organisations. This Wildlife Refuge is the only known breeding habitat of the Streaked Shearwater (*Calonectris leucomelas*) in Taiwan and also an important stopover site for migratory birds along the East Asian–Australasian Flyway. Furthermore, after livestock removal in 1996, secondary succession has been observed in plant taxa over the years.

Given the long-term accumulation of survey data associated with the Wildlife Refuge, we compiled and standardised data from historical reports and published the resulting dataset on the Global Biodiversity Information Facility (GBIF) under a CC BY 4.0 license. Before being made openly available, the dataset facilitated evidence-based conservation management and public policy development. Now, as an open-access dataset, it improves cross-border data integration and exchange. The dataset can support ecological research, while also improving public access to biodiversity information. This data paper describes Version 1.37 of the dataset. Please note that the dataset in the GBIF repository will be continuously updated as future annual surveys are completed.

**New information:**

This dataset compiles 20,533 occurrence records across nine taxonomic groups over this Wildlife Refuge from 1994 to 2025, allowing ecological succession to be examined. The species accumulation curves, plotted in survey order, show that the observed species richness for avian and plant taxa has reached a plateau, whereas the number of recorded species for other taxonomic groups continues to rise. Furthermore, the dataset highlights critical breeding events, such as the nests and juveniles of the Streaked Shearwater (*Calonectris leucomelas*), which were first recorded in 2010 and have been continuously documented through 2025. This finding makes this Wildlife Refuge the only known breeding habitat of the Streaked Shearwater in Taiwan. In addition to terrestrial and avian records, the ecological significance of this area extends to its marine environment as well; it encompasses an IUCN-designated Important Shark and Ray Area (ISRA) and documents globally significant species like *Manta birostris*, which is protected under Taiwan's Protected Marine Wildlife List.

## Introduction

### The history and geolocation of the Mianhua and Huaping Islets Wildlife Refuge

**Figure 1.**
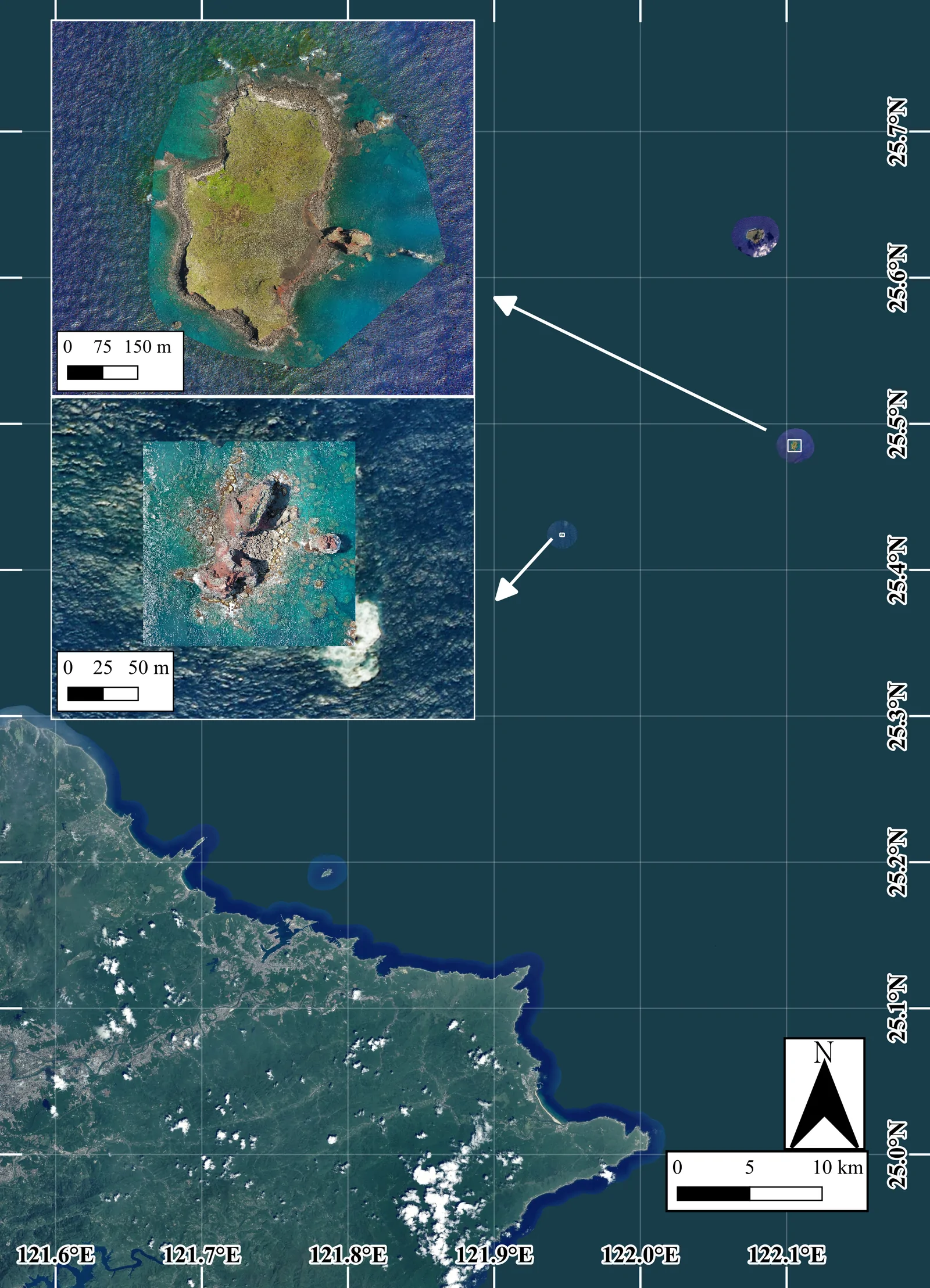
Location of Mianhua and Huaping Islets. The upper-left rectangle shows the Mianhua Islet and the lower-left rectangle shows the Huaping Islet. The basemap is an orthophotograph of mainland Taiwan provided by the National Land Surveying and Mapping Center.

**Figure 2.**
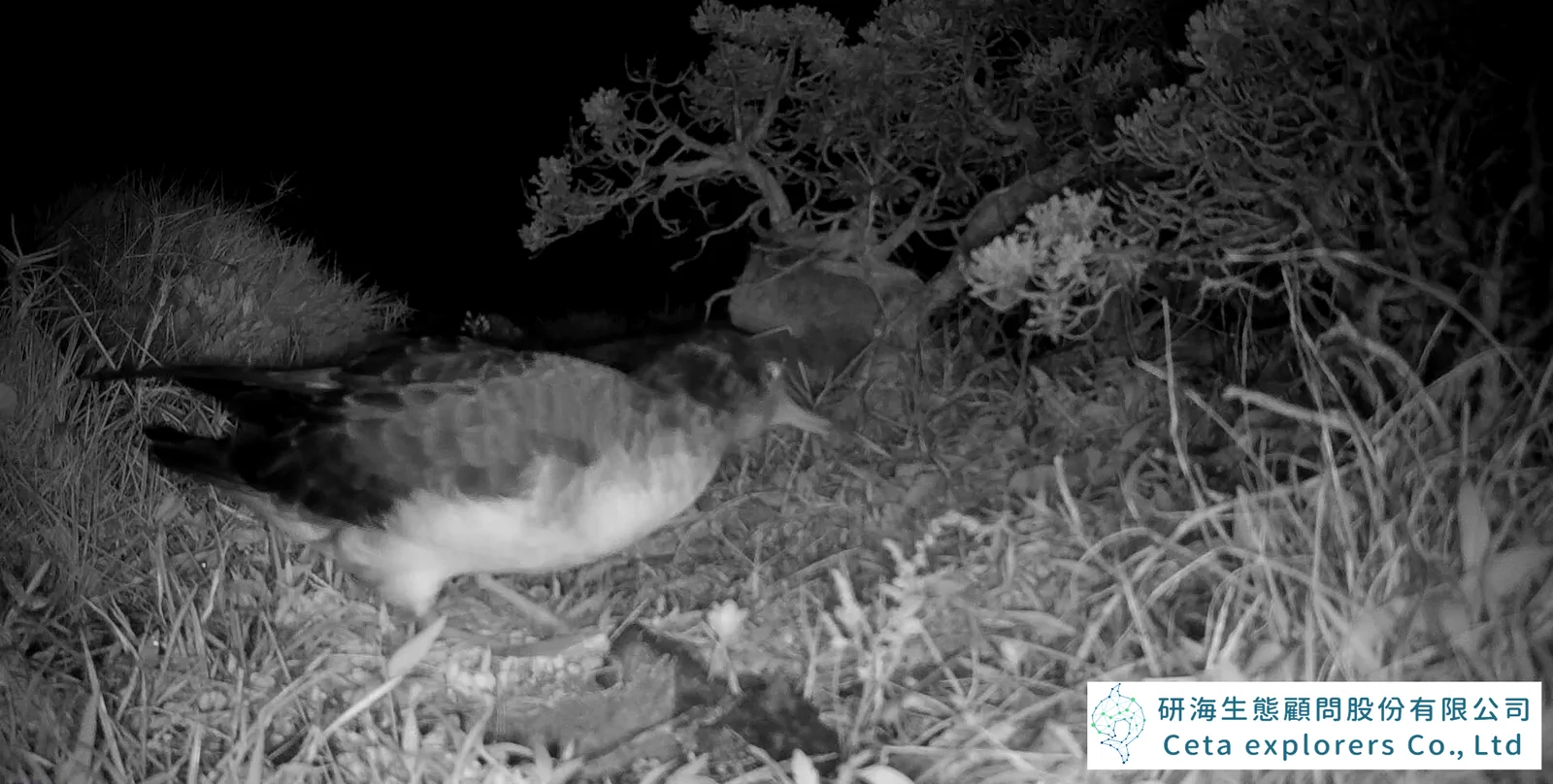
A Streaked Shearwate (*Calonectris leucomelas*) was recorded by a camera trap on 6 April 2024.

Mianhua Islet, Huaping Islet and Pengjia Islet are three volcanic offshore islets of Taiwan, located near the north-eastern territorial sea baseline (Fig. 1). In 1994, the Keelung City Government planned to develop these islets as a scenic area, yet the Wild Bird Society of Keelung raised concerns that it would cause huge ecological impacts (Chen-Chung Shen 1995). As a result, the Wild Bird Society of Keelung conducted the first documented ecological survey of avian and plant communities at Mianhua and Huaping Islets between May and September 1994, when the scenic draft plan was released. It was proposed to the central competent authority, the Council of Agriculture (now the Ministry of Agriculture), recommending that Mianhua and Huaping Islets be designated as a Nature Reserve. Finally, in 1996, the Council of Agriculture designated Mianhua and Huaping Islets as “Mianhua and Huaping Islets Wildlife Refuge”, rather than as a Nature Reserve. To eliminate disturbances from historical livestock farming, goats were removed from Mianhua Islet following the establishment of the Wildlife Refuge in 1996. Over the subsequent decades, the ecosystem has naturally recovered, characterised by a clear secondary succession in local plant taxa. Furthermore, in 2010, the Wild Bird Society of Keelung first recorded breeding nests of the Streaked Shearwater (*Calonectris leucomelas*). Since then, continuous occurrence records have been documented up to 2025 (Fig. 2). The Mianhua and Huaping Islets Wildlife Refuge is the only breeding habitat of the Streaked Shearwater in Taiwan. These birds nest in burrows near steep cliffs at the Wildlife Refuge, where they face predation threats from rats while breeding (Tsai et al. 2026). Due to an overall population decline, the IUCN Red List elevated the species' status from Least Concern to Near Threatened in 2015, a status that remains in effect. Rat-eradication work and breeding surveys at this Wildlife Refuge help maintain the Streaked Shearwater population. Although the refuge hosts a breeding colony of the Streaked Shearwater alongside resident species like the Peregrine Falcon (*Falco peregrinus*), the vast majority of avian species recorded are transient migrants. Situated along the East Asian–Australasian Flyway (EAAF), the refuge serves as a vital stopover before entering Taiwan, offering crucial resting and refuelling opportunities for migratory birds.

The central authority of the refuge was transferred from the Council of Agriculture to the Ocean Conservation Administration (OCA) under the Ocean Affairs Council (OAC) in April 2018. Subsequent regulatory amendments defining the spatial extent of the refuge and associated restrictions were announced by the Keelung City Government in July 2018. The Mianhua Islet is located at 25°29' N and 122°06' E, which covers 13.30 hectares. The Huaping Islet is located at 25°25' N and 121°56' E, which covers 3.08 hectares. The refuge was zoned into two regions: a core region comprising the terrestrial region of Mianhua and Huaping Islets (16.38 hectares) and a buffer region comprising the marine area (210 hectares). The buffer region extends 500 m from the Mean Low Water Line of the Mianhua Islet (188 hectares) and extends 200 m from the Mean Low Water Line of the Huaping Islet (22 hectares). Although the total area of the refuge is 226.38 hectares, the spatial coverage of this survey data extends beyond the legally defined refuge boundary, particularly in the avian and cetacean surveys.

Since 2008, the ecology surveys have been commissioned as annual local government projects when funding is available for tenders. Multiple organisations may bid to serve as the implementing group if they meet the selection criteria and the final implementing group is selected by the Procurement Evaluation Committee.

### Species Accumulation Curves Represent Species Richness

Sample-based species accumulation curves (SAC) were used to evaluate species richness in relation to survey effort (Gotelli and Colwell 2001). Given the restricted access and uninhabited status of the Wildlife Refuge, field surveys are logistically constrained to maritime transport and require prior governmental approval. The public release of this long-term dataset provides a taxonomically comprehensive record spanning more than three decades and supports comparative studies of species richness and community composition on volcanic islets within the Ryukyu Arc, part of the East Asian Island Arc.

## Project description

### Title

The Patrol and Biological Survey of Mianhua and Huaping Islets Wildlife Refuge

### Personnel

**Figure 3.**
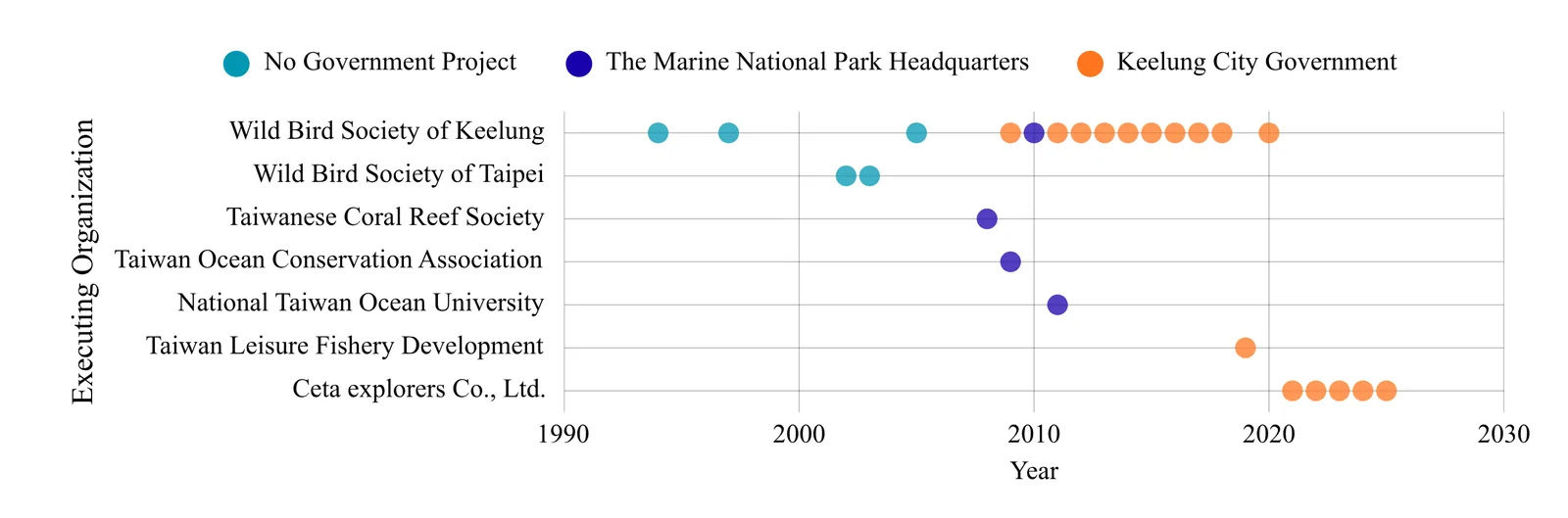
Timeline of ecological surveys (1994–2025), detailing executing organisations on the y-axis and their respective funding agencies by marker colour.

The personnel and administration of the surveys can be classified into three distinct stages, based on funding and management (Fig. 3, Suppl. material 1). Throughout the entire survey period from 1994 to 2025, the Local Competent Authority has consistently been the Keelung City Government. Meanwhile, the Central Competent Authority was the Forestry and Nature Conservation Agency under the Ministry of Agriculture from 1994 to 2018 and shifted to the Ocean Conservation Administration under the Ocean Affairs Council from 2019 to 2025.

1. **Before the survey became a government project (1994–2005)**: Before formal government-funded survey projects were established, ecological surveys of the Wildlife Refuge were conducted by civil society organisations. The Wild Bird Society of Keelung surveyed the refuge in 1994, 1997 and 2005 and the Wild Bird Society of Taipei conducted surveys in 2002 and 2003 (Keelung Wild Bird Society 2012).
2. **Early government-funded surveys (2008–2011)**: From 2008 to 2011, surveys entered an early government-funded phase, mainly administered by the Marine National Park Headquarters, Ministry of the Interior. These surveys were conducted by the Taiwanese Coral Reef Society in 2008, by the Taiwan Ocean Conservation Association and the Wild Bird Society of Keelung in 2009 and by the Wild Bird Society of Keelung in 2010. In 2011, the Marine National Park Headquarters commissioned National Taiwan Ocean University to conduct the marine survey, while the Keelung City Government commissioned the Wild Bird Society of Keelung to conduct the terrestrial survey (Chang-Feng Tai et al. 2008, Ching-Ta Chuang and Man-Se Chan 2009, Chen-Chung Shen et al. 2010, I-Hsiung Chen et al. 2011, Yueh-Tzu Yang et al. 2011).
3. **Full administration by the Keelung City Government (2012–2025)**: From 2012 to 2025, survey projects were administered by the Keelung City Government and implemented by different organisations across years. The implementing organisations include: the Wild Bird Society of Keelung, which conducted surveys from 2012 to 2018 and in 2020; the Taiwan Leisure Fishery Development, which surveyed in 2019; and Ceta explorers Co., Ltd., which surveyed from 2021 to 2025 (Keelung Wild Bird Society 2012, Keelung Wild Bird Society 2013, Keelung Wild Bird Society 2014, Chen-Chung Shen et al. 2015, Yu-Chien Chen 2015, Chen-Chung Shen et al. 2016, Chen-Chung Shen et al. 2017, Chin-Feng Shen 2018, Cheng-Wei Tso 2019, Chen-Chung Shen et al. 2020, Yen-Ting Huang and Chung-Hang Hung 2021, Yen-Ting Huang and Chung-Hang Hung 2022, Yen-Ting Huang and Chung-Hang Hung 2023, Yen-Ting Huang and Chung-Hang Hung 2024, Yen-Ting Huang and Chung-Hang Hung 2025).

### Study area description

The study area of the terrestrial region always includes the Mianhua Islet and Huaping Islet, whereas Pengjia Islet is included only in a few years. The study area of the marine region includes the route between Bachimen Fishing Harbour and the Mianhua and Huaping Islets Wildlife Refuge, as well as the surrounding water.

### Design description

“The Patrol and Biological Survey of Mianhua and Huaping Islets Wildlife Refuge” is a government-commissioned project designed to monitor ecological changes in the Wildlife Refuge, assess potential human impacts and support management strategies. The earliest survey records date back to 1994, two years before the Wildlife Refuge was established in 1996. Surveys were conducted intermittently before 2008 and annually thereafter through 2025 by different implementing organisations. The project is currently administered and commissioned by the Keelung City Animal Protection and Health Inspection Office under the Keelung City Government.

### Funding

From 1994 to 2005, there was no designated government project funding. In 2008 and 2010, the ecological surveys were funded solely by the Marine National Park Headquarters, under the Ministry of the Interior. In 2009 and 2011, the ecological survey was jointly funded by the Marine National Park Headquarters and by the Keelung City Government. From 2012 to 2025, all survey activities were primarily funded by the Keelung City Government, with occasional subsidies from the central government.

## Sampling methods

### Study extent

This dataset covers ecological surveys conducted between 1994 and 2025, with survey years including 1994, 1997, 2002–2003, 2005 and 2008–2025. The records encompass nine taxonomic groups: algae, avians, benthic invertebrates, fish, plants, reptiles, insects, spiders and cetaceans, with cetacean surveys beginning in 2019. Data were compiled from survey reports produced by different implementing organisations, as well as journal articles and newsletters. Sampling areas included the route from Bachimen Fishing Harbour to the Mianhua and Huaping Islets Wildlife Refuge and the surrounding waters. Additional terrestrial bird surveys were conducted on Pengjia Islet by the Wild Bird Society of Keelung.

### Sampling description

Sampling methods varied amongst taxonomic groups, which were classified into nine categories: avians, cetaceans, benthic invertebrates, fish, plants, insects, spiders, algae and reptiles.

### Quality control

For records from 1994 to 2020, data were compiled from published reports for which raw data were unavailable; quality control, therefore, relied on the procedures applied by the original survey teams. Since 2021, quality control has been conducted by the present project team, Ceta explorers Co., Ltd., following a standardised operating procedure (SOP), including:

1. Before the surveys:
  - Confirm that the surveyors are experienced in species identification.
2. During the surveys:
  - Except for machine observations, ensure there are no duplicate sightings at the individual level.
3. After the surveys:
  - Check that the GPS coordinate records correspond to the correct zone.
  - For records lacking a precise GPS point, the Point-Radius Method was applied. The exact centroid of the respective locality (using 5 decimal places, for example, coordinatePrecision = 0.00001) was assigned, while the actual spatial accuracy and fuzziness of the observation are represented by the coordinateUncertaintyInMeters (the Radius). The coordinateUncertaintyInMeters for the nine taxonomic groups are listed in Table 2. For records without exact GPS tracks, the following centroids were used:
    - Terrestrial organism at Mianhua Islet: (25.48483, 122.10535)
    - Marine organism around Mianhua Islet: (25.48483, 122.10272)
    - At Huaping Islet: (25.42414, 121.94638)
    - At Pengjia Islet: (25.62849, 122.07898)
    - At Waters off Keelung: (25.37926, 121.96444)
  - Except for machine observations, verify that there are no duplicate sightings at the individual level.
  - The spelling and biological classification of the scientific names were validated by NomenMatch and Catalogue of Life in Taiwan (TaiCOL), which are scientific-name checking tools developed by the Taiwan Biodiversity Information Facility (TaiBIF).
  - Before the dataset publication, the data format and structure were corrected by the GBIF data validator, which was developed by the GBIF Secretariat.

### Step description

The Sampling methods were classified into these nine categories: avians, cetaceans, benthic invertebrates, fish, plants, insects, spiders, algae and reptiles. The methods were listed as follows (Table 1).

**Figure 4.**
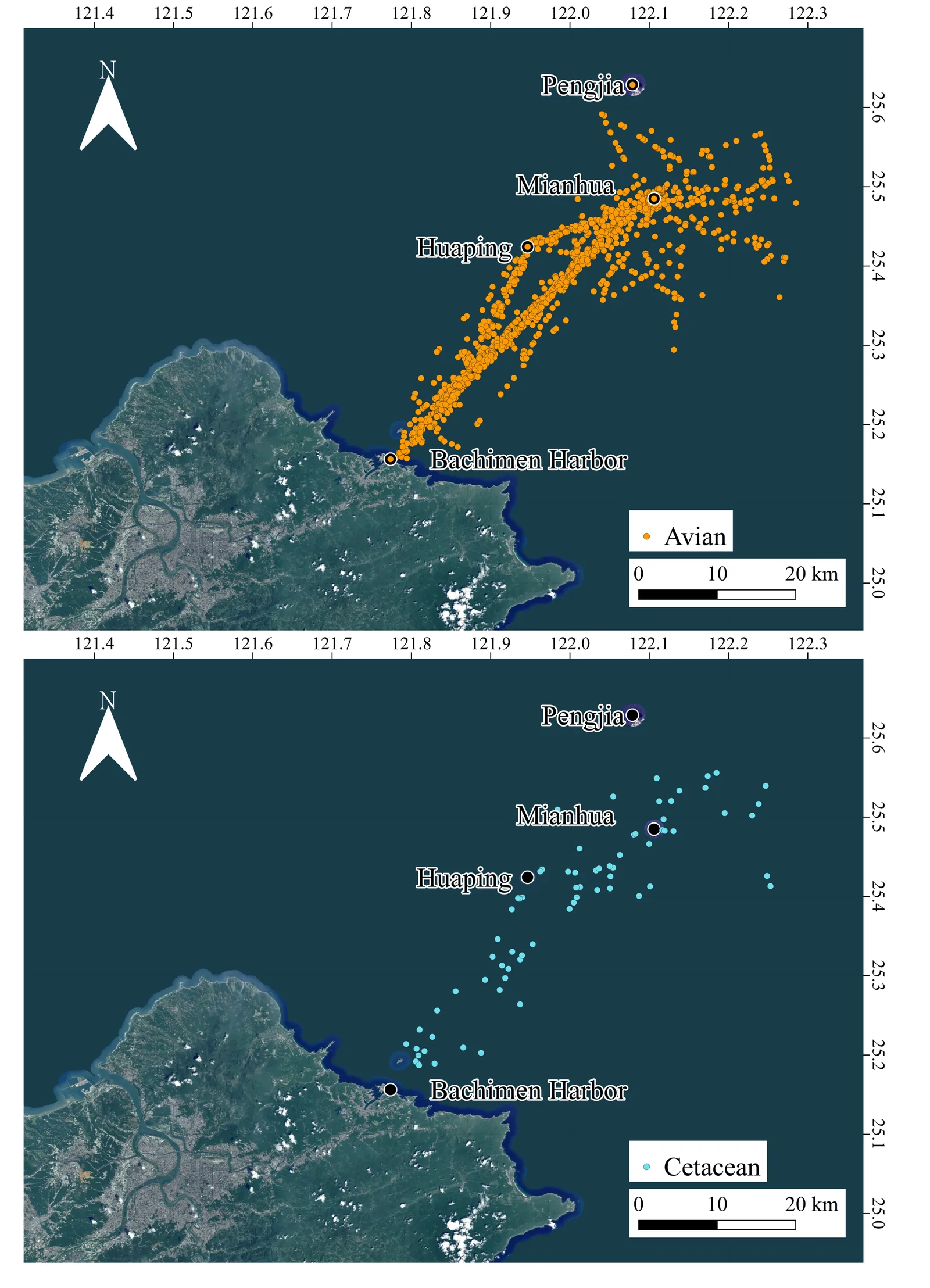
The upper image shows the distribution of avian occurrences in this dataset from 1994 to 2025; the lower image shows the distribution of cetacean occurrences in this dataset from 2019 to 2025. The location of the Mianhua, Huaping, Pengjia Islets and the Bachimen Harbour is specifically labelled. The basemap is an orthophotograph of Taiwan provided by the National Land Surveying and Mapping Center.

**Figure 5.**
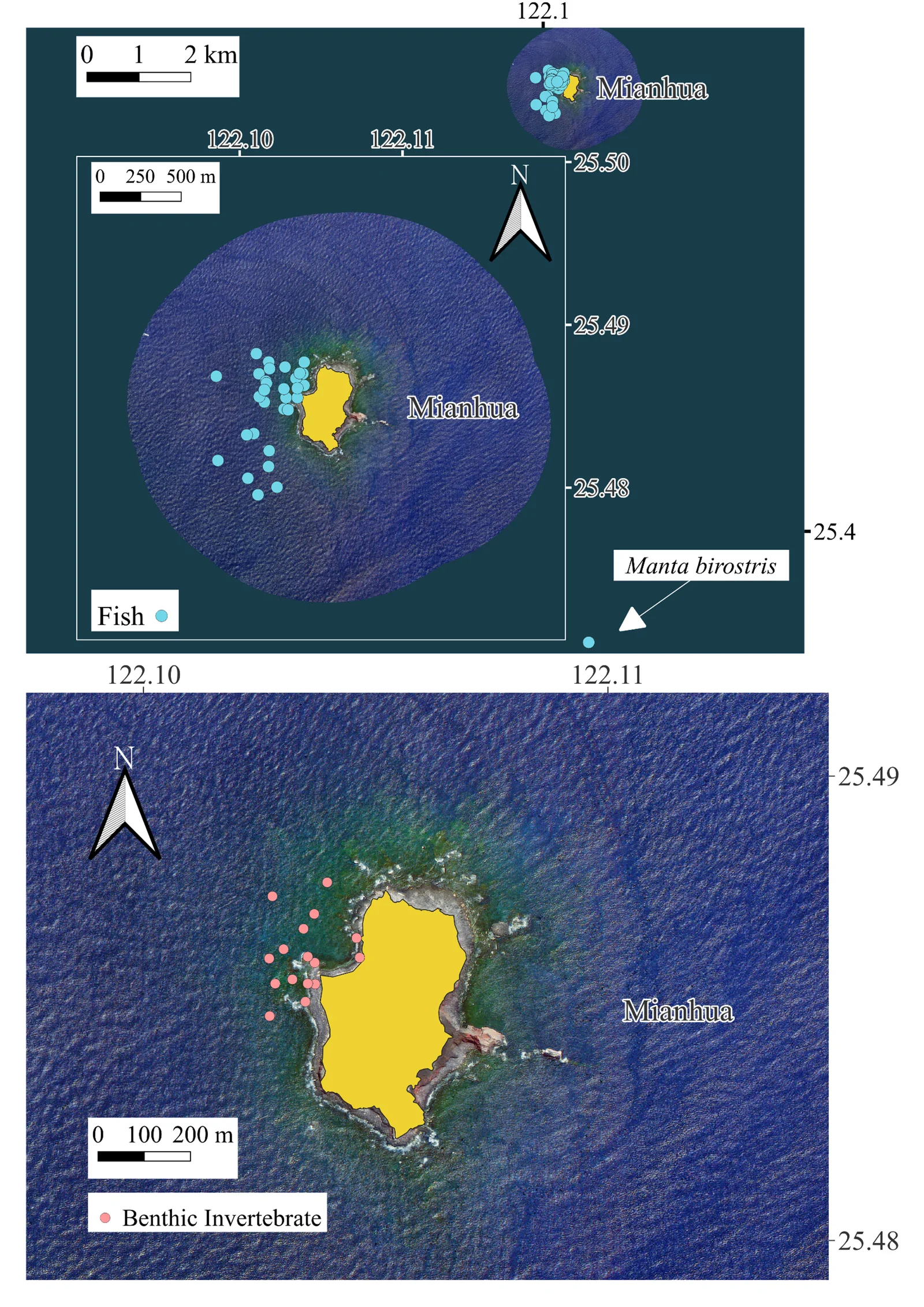
The upper image shows the distribution of fish occurrences in this dataset in 2014, 2017–2018, 2020 and 2022–2025; the lower image shows the distribution of benthic invertebrate occurrences in this dataset in 2011 and 2020–2025. The location of the *Manta birostris* sighted on 4 June 2022 is specifically labelled. The basemap is an orthophotograph of Mianhua Islet provided by the National Land Surveying and Mapping Center.

**Figure 6.**
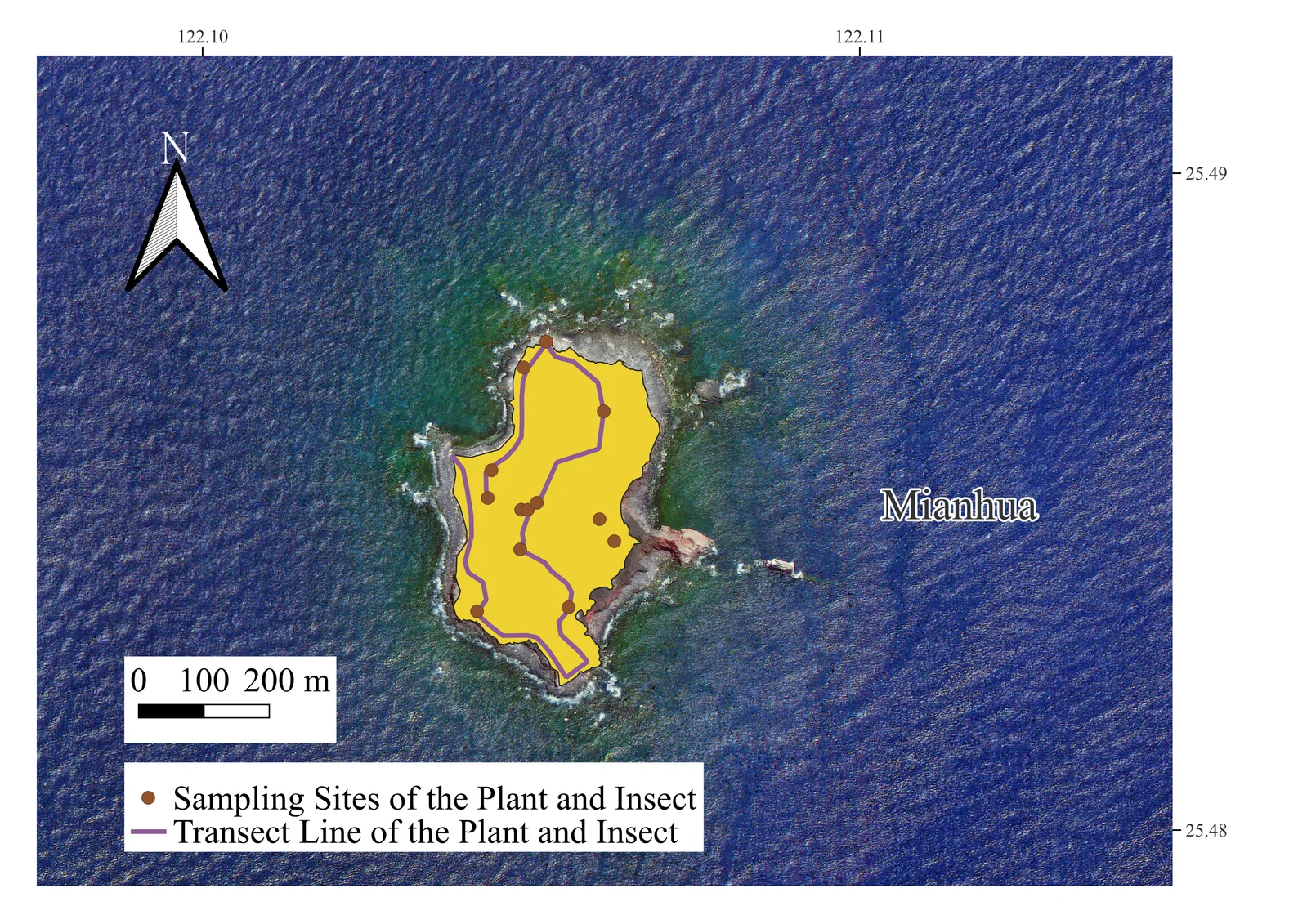
The image shows the transect line and sampling sites of the plant and insect. The basemap is an orthophotograph of Mianhua Islet provided by the National Land Surveying and Mapping Center.

1. **The Avian Survey**: The avian survey was based on visual observations conducted on land at Mianhua or Pengjia Islets and at the surrounding waters of Mianhua, Huaping and Pengjia Islets. Sightings were also recorded along the patrol routes, including the round-trip route between Bachimen Fishing Harbour and Mianhua and Huaping Islets (Fig. 4). The surveys were conducted in 1994, 2002–2003, 2005 and 2008–2025. In addition, trail cameras were deployed on Mianhua Islet from 2022 for additional data collection.
2. **The Cetacean Survey**: Since 2019, cetacean sightings have been recorded along the patrol routes, including the round-trip route between Bachimen Fishing Harbour and Mianhua and Huaping Islets (Fig. 4).
3. **The Fish Survey**: Fish surveys used the strip-transect method by Self-Contained Underwater Breathing Apparatus (SCUBA) Diving (30 m * 5 m) in 2011. In 2020-2025, the surveys used the Underwater Visual Census (UVC), the roving diver method, Baited Remote Underwater Video (BRUV), Diver-Operated Video (DOV) and occasional sightings during the patrol (Fig. 5).
4. **The Benthic Invertebrates Survey at Mianhua Islet**: The methods for benthic invertebrates included snorkelling, SCUBA diving and intertidal qualitative surveys (Fig. 5). The surveys were conducted in 2014, 2017–2018, 2020 and 2022–2025.
5. **The Plant Survey at Mianhua Islet**: The surveys were conducted in 1994, 1997, 2005, and 2008–2023. Surveys were conducted using grid sampling or the line transect method from 2008 and from 2011 to 2023 (Fig. 6). The grid sampling consisted of thirteen sample plots distributed across Mianhua Islets and their central coordinates are listed in Table 3. The survey methods in other years were either undocumented or varied. During these surveys, species identification and coverage were recorded.
6. **The Insect Survey at Mianhua Islet**: The insect surveys were conducted by line transect, sweep netting, trapping, baiting, aerial netting, beat sheet sampling, sieving, aquatic netting, hand searching, visual searches and host plant identification (Fig. 6). The surveys were conducted in 1994 and 2008–2025.
7. **The Algae Survey at Mianhua Islet**: Algae were surveyed by snorkelling or by an intertidal qualitative survey. Algae were not a primary target of the project.
8. **The Reptile Survey at Mianhua Islet**: The reptile survey on Mianhua Islet was conducted through a line transect survey or opportunistic sightings. Reptiles were not a primary target of the project. The occurrence records were in 2008-2017 and 2020-2025.
9. **The Spider Survey at Mianhua Islet**: In 2008, the spider survey on Mianhua Islet was conducted through opportunistic sightings.

## Geographic coverage

### Description

The data covered the sea area and the islets of northern Taiwan, which include Mianhua Islet, Huaping Islet, Pengjia Islet and the surrounding waterbody (Fig. 4).

### Coordinates

25.156° N and 25.649° N Latitude; 121.772° E and 122.294° E Longitude.

## Taxonomic coverage

### Description

The checklist of this dataset includes nine taxonomic groups: avians, cetaceans, benthic invertebrates, fish, plants, insects, spiders, algae and reptiles. The algae, spiders and reptiles are excluded from the species accumulation curves because these groups are not the primary survey targets in this long-term dataset. The sample-based species accumulation is chosen because occurrence counts were not recorded in the early survey reports; only taxa were recorded. The accumulation curve is analysed at the species level, so all individuals identified in higher taxa are filtered out and individuals identified at the subspecies level are reclassified to the species level. The accumulation curves were plotted using Python 3.12. The table of the six groups on which the species accumulation curves were plotted is shown in Suppl. material 2. The code and supplementary tables used for data cleaning, standardisation, analysis and figure generation are available on GitHub and archived on Zenodo (JuiWenChang 2026) under doi: https://doi.org/10.5281/zenodo.23238663.

**Figure 7.**
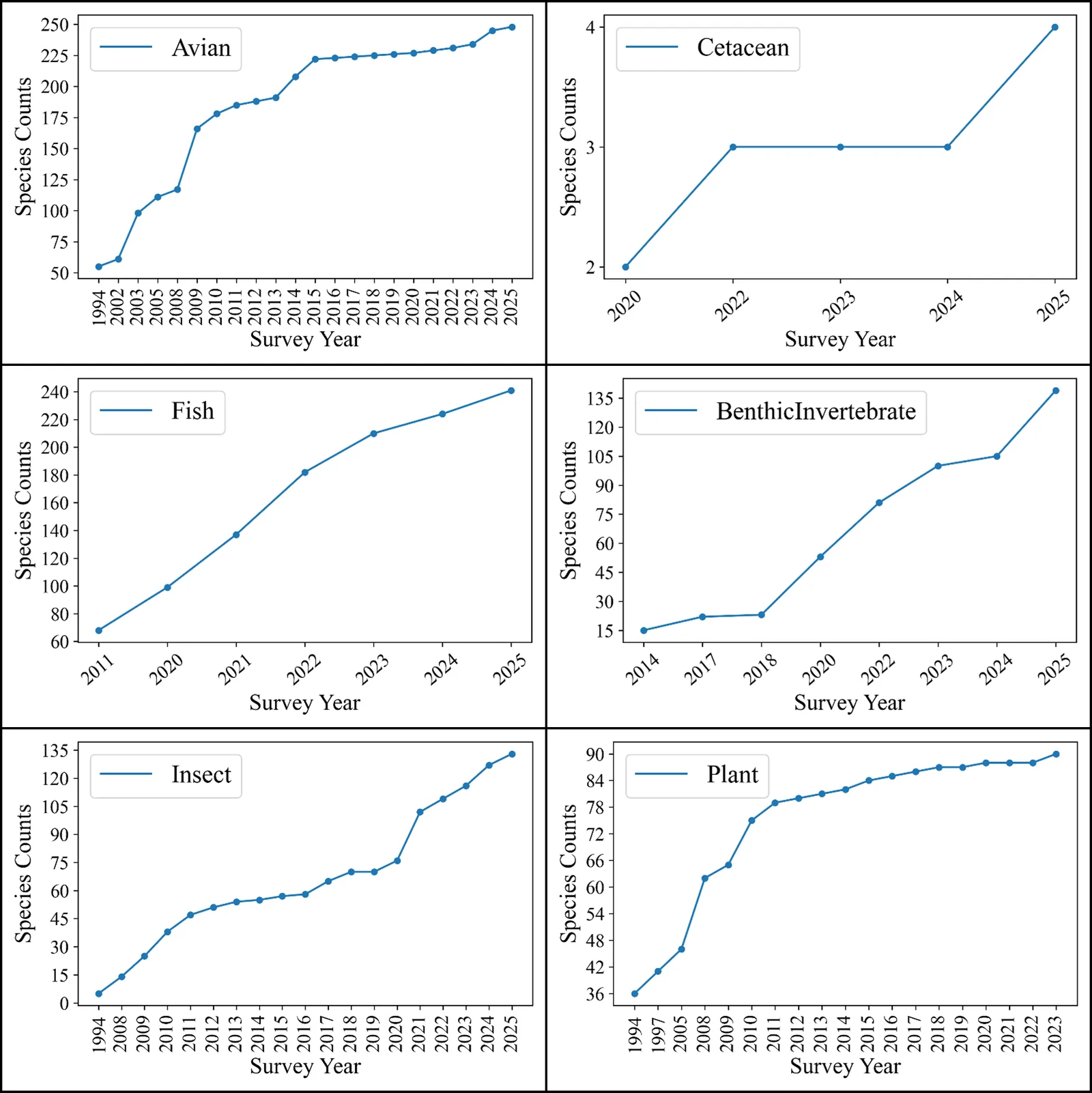
Species accumulation curves of six taxonomic groups. The x–axis shows the survey years and the y–axis shows the cumulative number of taxa identified to the species level.

The sample-based species accumulation curve is the cumulative species counts over the survey year in the original survey order (Fig. 7). Based on the flattening slopes of the species accumulation curves, the number of observed species for avian and plant taxa has largely plateaued, while new records for other groups are still accumulating.

**Figure 8.**
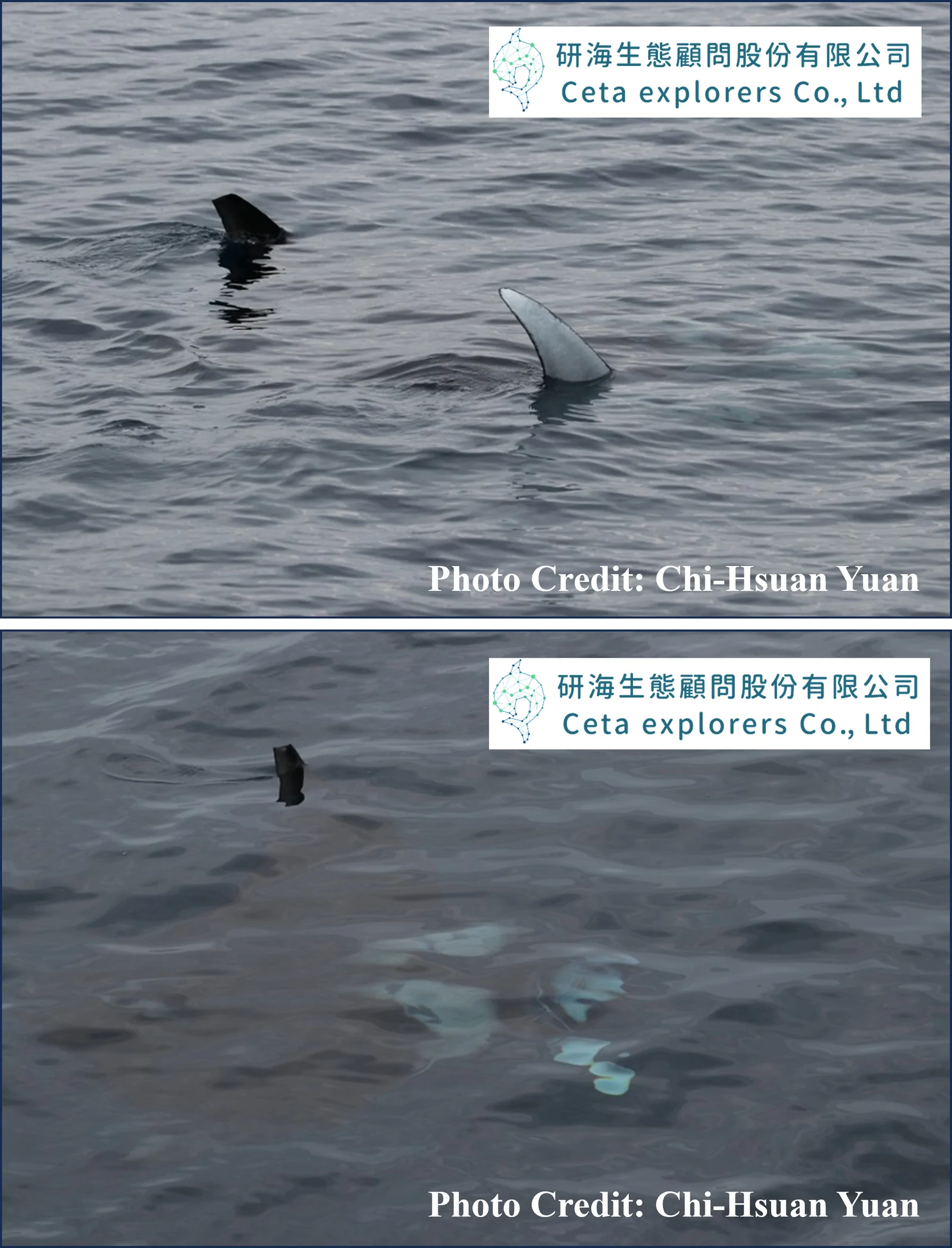
The *Manta birostris* was opportunistically sighted along the round-trip route between Bachimen Fishing Harbour and the Mianhua and Huaping Islets Wildlife Refuge. The upper and lower images show the same individual at different moments during swimming.

The marine area of this refuge overlaps with one of the Important Shark and Ray Areas (ISRA) designated by the International Union for Conservation of Nature (IUCN). This ISRA is named “Turtle–Northern Islands ISRA”, extending from Gueishan (Turtle) Island in the south to these three volcanic islets in the north. In this dataset, Chondrichthyan species have been recorded through visual observation and Baited Remote Underwater Video (BRUV) in 2011 and 2022–2025. Most of the Chondrichthyan species recorded by BRUV were identified at the family level due to the limitations in video resolution, so these records were excluded from the species accumulation curve. On 4 June 2022, *Manta birostris* was sighted and it is in the Protected Marine Wildlife List of Taiwan (Figs 5, 8).

### Taxa included

**Table:** 

Rank	Scientific Name	Common Name
kingdom	Animalia	Animals
kingdom	Plantae	Plants
phylum	Chlorophyta	Green Algae
phylum	Rhodophyta	Red Algae
phylum	Tracheophyta	Vascular Plants
phylum	Annelida	
phylum	Mollusca	
phylum	Cnidaria	
phylum	Echinodermata	
phylum	Arthropoda	
phylum	Chordata	
class	Chondrichthyes	Sharks and Rays or Fish
class	Osteichthyes	Fish
class	Insecta	Insects
class	Arachnida	Spiders
class	Reptilia	Reptiles
class	Aves	Birds
class	Mammalia	Mammals
infraorder	Cetacea	Whales and Dolphins

## Temporal coverage

**Data range:** 1994-1-01 – 2025-11-26.

### Notes

The earliest record in the Mianhua and Huaping Islets Wildlife Refuge dates back to 1994 and the ecological survey continues running now. Spanning over three decades from 1994 to 2025, this dataset comprises 23 years of intermittent survey records. The times of the surveys differ between various survey targets. The main targets are: avians, cetaceans, benthic invertebrates, fish, plants and insects. This long-period dataset allows ecological succession to be examined, especially in the plant group.

**Figure 9.**
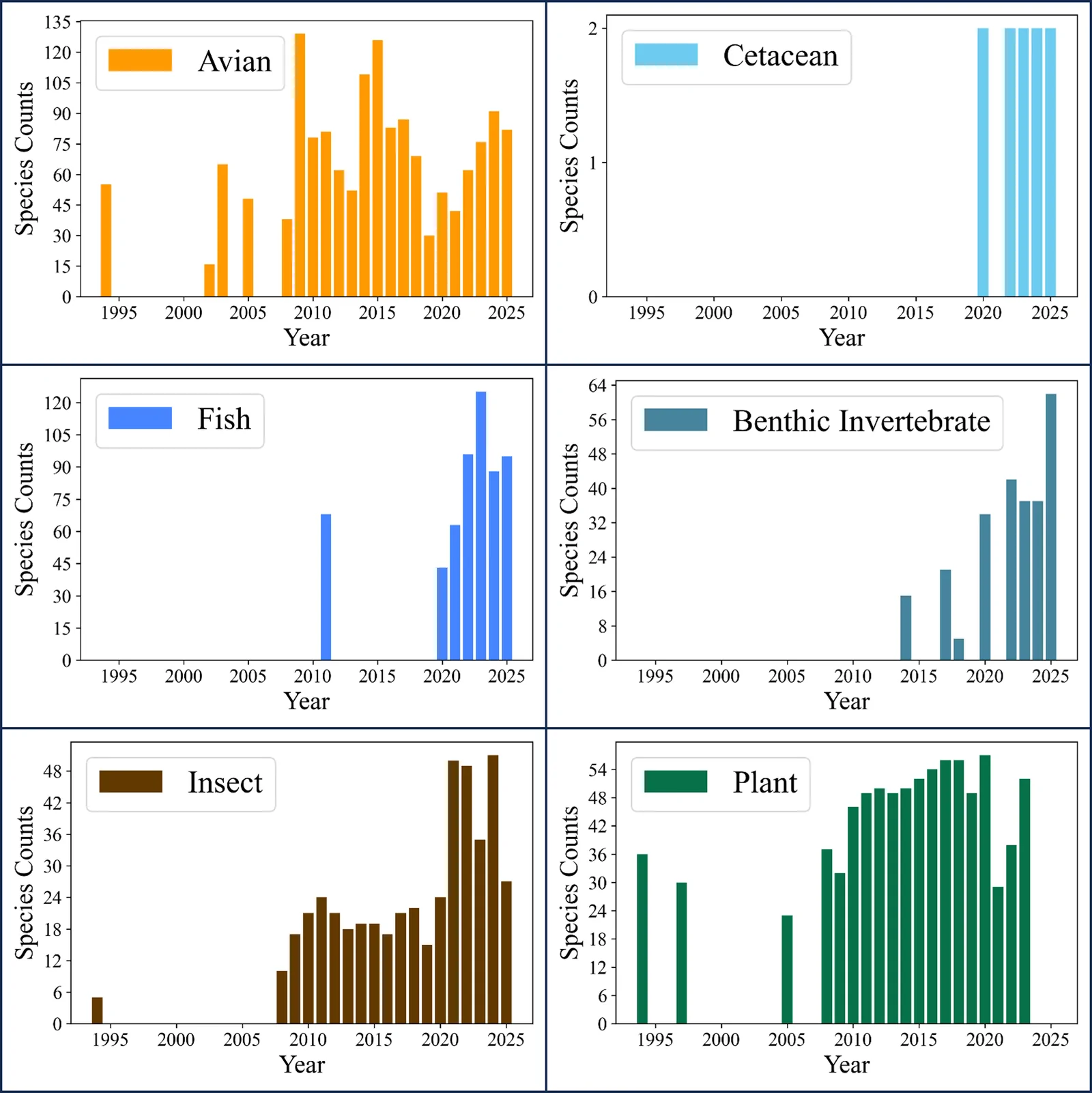
Annual species counts for six taxonomic groups. Years without bars indicate that no surveys were conducted or no sightings were recorded for that taxonomic group. The x–axis shows survey years from 1994 to 2025 and the y–axis shows the number of taxa identified to the species level in each survey year.

In the early survey reports, only taxa were recorded and occurrence counts were not recorded. Accordingly, annual occurrence counts were not analysed and annual taxon counts of the six primary survey targets are presented in Fig. 9. Years without bars indicate that no surveys were conducted or no sightings were recorded for that taxonomic group. The table of the six taxonomic groups on which annual species counts were plotted is shown in Suppl. material 3.

## Usage licence

### Usage licence

Open Data Commons Attribution License

### IP rights notes

This work is licensed under a Creative Commons Attribution (CC-BY) 4.0 License.

## Data resources

### Data package title

The survey data of Mianhua and Huaping Islets Wildlife Refuge

### Resource link

https://doi.org/10.15468/vednhh

### Alternative identifiers

https://ipt.taibif.tw/resource?r=cetaexplorers_mianhua_huaping_islets&v=1.37

### Number of data sets

2

### Data set 1.

#### Data set name

Occurrence

#### Data format

Darwin Core standard (DwC)

#### Character set

UTF-8

#### Data format version

1.37

#### Description

Darwin Core is an evolving, community-developed biodiversity data standard (Wieczorek et al. 2012). This dataset follows the Darwin Core standard (DwC). The data model of this dataset is a star schema, consisting of one core table and multiple extension tables linked to it. In this dataset, the core table is occurrence-based and covers nine taxonomic groups: algae, avians, benthic invertebrates, cetaceans, fish, plants, reptiles, spiders and insects. A single extension table is included, an Extended Measurement or Fact table, which records the body length measurement for the fish group.

The first occurrence record dates back to 1994, although surveys were not conducted on an annual basis until 2008. Prior to 2008, the study primarily focused on terrestrial ecology, such as avians and plants, whereas insect surveys were incorporated in later years. In 2018, the jurisdiction over the refuge was transferred from the National Agriculture Administration to the Ocean Conservation Administration (OCA) under the Ocean Affairs Council (OAC). Following this administrative change, greater emphasis was placed on aquatic ecology, including the benthic invertebrates, fish and cetaceans. Surveys of algae, spiders and reptiles were not the primary study targets. The algae survey was seldom conducted, while reptile and spider records were derived from opportunistic sightings during other survey activities.

The required attributes of the occurrence-based core table include event date, locality, scientific name, individual count, basis of record and a unique occurrence ID. Column names of the core table are consistent across the nine organism groups, resulting in a total of 41 columns, yet only the marine species (e.g. fish, benthic invertebrates, cetaceans) have values in “minimumDepthInMeters” and “maximumDepthInMeters”. As fish records are collected at the individual level, the extension table focused on measurements of the total length. In this "extendedmeasurementorfact" table, body length measurements of fish surveys were exclusively collected using the Underwater Visual Census (UVC) Line Transect method; therefore, Baited Remote Underwater Video (BRUV) or Roving Diver Method surveys were not included. The core and extension tables are linked by the occurrence ID. The column labels and descriptions are in Darwin Core format, as listed in the following table. The column definitions follow the List of Darwin Core terms (Darwin Core Maintenance Group 2025). The dataset is published on the Global Biodiversity Information Facility (GBIF) and all users can download the dataset in TXT format (Chang et al. 2026).

**Data set 1.** 

Column label	Column description
taxonID	An identifier for the set of Taxon information. It is an identifier specific to the dataset.
occurrenceID	A unique identifier for the Occurrence record.
eventDate	The date-time when the Event was recorded.
year	The four-digit year in which the Event occurred, according to the Common Era Calendar.
month	The integer month in which the Event occurred.
day	The integer day of the month on which the Event occurred.
eventTime	The time or interval during which an Event occurred.
basisOfRecord	The specific nature of the data record.
continent	The name of the continent in which the Location occurs.
country	The name of the country or major administrative unit in which the Location occurs.
locality	The specific description of the place.
samplingProtocol	The names of, references to, or descriptions of the methods or protocols used during an Event.
scientificName	The full scientific name, with authorship and date information if known. When forming part of an Identification, this should be the name in the lowest level taxonomic rank that can be determined.
taxonRank	The taxonomic rank of the most specific name in the scientificName.
vernacularName	A common or vernacular name.
individualCount	The number of individuals present at the time of the Occurrence.
organismQuantityType	The type of quantification system used for the quantity of Organisms.
organismQuantity	A number or enumeration value for the quantity of Organisms.
georeferenceVerificationStatus	A categorical description of the extent to which the georeference has been verified to represent the best possible spatial description for the Location of the Occurrence.
geodeticDatum	The ellipsoid, geodetic datum or spatial reference system (SRS) upon which the geographic coordinates given in decimalLatitude and decimalLongitude are based.
decimalLongitude	The geographic longitude (in decimal degrees, using the spatial reference system given in geodeticDatum) of the geographic center of a dcterms:Location. Positive values are east of the Greenwich Meridian, negative values are west of it. Legal values lie between -180 and 180, inclusive.
decimalLatitude	The geographic latitude (in decimal degrees, using the spatial reference system given in geodeticDatum) of the geographic center of a dcterms:Location. Positive values are north of the Equator, negative values are south of it. Legal values lie between -90 and 90, inclusive.
coordinatePrecision	A decimal representation of the precision of the coordinates given in the decimalLatitude and decimalLongitude.
coordinateUncertaintyInMeters	The horizontal distance (in meters) from the given decimalLatitude and decimalLongitude describing the smallest circle containing the whole of the Location.
recordedBy	A person, group or organisation responsible for recording the original Occurrence.
kingdom	The full scientific name of the kingdom in which the Taxon is classified.
reproductiveCondition	The reproductive condition of the biological individual(s) represented in the Occurrence.
behavior	The behavior shown by the subject at the time the Occurrence was recorded.
occurrenceRemarks	Comments or notes about the Occurrence.
id	A system-generated core identifier created by the Global Biodiversity Information Facility (GBIF) to ensure each record is uniquely identified in an aggregated download. While occurrenceID is unique within this specific dataset, the id column prevents duplication when combining records across different datasets globally.
associatedReferences	A list (concatenated and separated) of identifiers (publication, bibliographic reference, global unique identifier, URI) of literature associated with the Occurrence.
dynamicProperties	A list of additional measurements, facts, characteristics, or assertions about the record. Meant to provide a mechanism for structured content.
identificationQualifier	A brief phrase to express the determiner's doubts about the dwc:Identification.
verbatimIdentification	A string representing the taxonomic identification as it appeared in the original record.
countryCode	The standard code for the country in which the locality occurs.
minimumDepthInMeters	The lesser depth of a range of depths below the local surface, in meters.
maximumDepthInMeters	The greater depths of a range of depths below the local surface, in meters.
taxonConceptID	An identifier for the taxonomic concept to which the record refers.
taxonomicStatus	The status of the use of the dwc:scientificName as a label for a taxon. It must be linked to a specific taxonomic reference that defines the concept.
scientificNameID	An identifier for the nomenclatural details of a scientific name, as opposed to its taxonomic details.
taxonRemarks	Comments or notes about the taxon or name.

### Data set 2.

#### Data set name

extendedMeasurementOrFacts

#### Data format

Darwin Core standard (DwC)

#### Character set

UTF-8

#### Data format version

1.37

**Data set 2.** 

Column label	Column description
measurementID	An identifier for the dwc:MeasurementOrFact (information pertaining to measurements, facts, characteristics or assertions). May be a global unique identifier or an identifier specific to the dataset.
id	A system-generated core identifier created by the Global Biodiversity Information Facility (GBIF) to ensure each record is uniquely identified in an aggregated download. While occurrenceID is unique within this specific dataset, the id column prevents duplication when combining records across different datasets globally.
occurrenceID	A unique identifier for the Occurrence record.
measurementDeterminedDate	The date on which the dwc:MeasurementOrFact was made.
measurementType	The nature of the measurement, fact, characteristic or assertion.
measurementValue	The value of the measurement, fact, characteristic or assertion.
measurementUnit	The units associated with the measurementValue.
measurementDeterminedBy	A person, group or organisation who determined the value of the dwc:MeasurementOrFact.
measurementRemarks	Comments or notes accompanying the dwc:MeasurementOrFact.

## Additional information

With the use of the dataset:

1. There are 1070 unique values in the scientificName, whereas there are 1072 unique values in taxonID and verbatimIdentification (one-to-one relationship). This discrepancy arises because the genus *Phylloscopus* corresponds to three different taxonIDs in our database due to indistinguishable species complexes in the field. Specifically: One taxonID represents the Arctic Warbler complex (*Phylloscopus xanthodryas/borealis/examinandus*). Another taxonID represents the Blyth's/Claudia's/Hartert's Leaf Warbler complex (*Phylloscopus reguloides/claudiae/goodsoni*). The third taxonID is used for general *Phylloscopus* species that could not be assigned to either complex. To adhere to DwC, the scientificName was kept at the genus level (*Phylloscopus*) for all three. However, the ambiguity has been resolved by adding the species complex details into the "vernacularName", "verbatimIdentification", "identificationQualifier" and "taxonRemarks" fields.
2. Similarly, the taxonID 'KL_Avian_0000165' represents the Japanese bush warbler complex in broad terms (*Horornis diphone*/*canturians*), while its scientificName is recorded at the genus level, Horornis. Although the genus Horornis encompasses four species in total, records of the generic Horornis species that could not be assigned to any complex are not present in our current dataset.
3. Cetaceans in mixed-species groups have separate occurrence-level sighting records.

## Supplementary Material

86B65813-6FB0-5C30-9A51-D50B7E6580B7 10.3897/BDJ.14.e209979.suppl1 Supplementary material 1 Administrative details of the ecological surveys conducted from 1994 to 2025 Data type table Brief description Administrative details of the ecological surveys conducted from 1994 to 2025. This table serves as the underlying data for Fig. 3, detailing the four administrative roles associated with each survey year: executing organisation, funding agency, local competent authority and central competent authority. File: oo_1777684.csv https://binary.pensoft.net/file/1777684 Jui-Wen, Chang

954CF56D-2EB0-5A45-85D4-23999D7F3C85 10.3897/BDJ.14.e209979.suppl2 Supplementary material 2 Table of yearly species accumulation curves for six taxonomic groups from 1994 to 2025 Data type species accumulation curve data Brief description The yearly species accumulation curves of six taxonomic groups (Fig. 7) were plotted, based on this table. The first column is the table index and lists survey years from 1994 to 2025, annually. The first row contains the column headers corresponding to the six groups. A null value indicates that no surveys were conducted or no sightings were recorded for that taxonomic group in that year. The integer values indicate the cumulative number of species recorded from 1994 to the year specified in each row for the corresponding taxonomic group. File: oo_1789276.csv https://binary.pensoft.net/file/1789276 Jui–Wen Chang

5D409765-00CC-5B77-AC9C-4E6F8DE3D7B8 10.3897/BDJ.14.e209979.suppl3 Supplementary material 3 Table of annual species counts for six taxonomic groups from 1994 to 2025 Data type annual species counts data Brief description The annual species counts for six taxonomic groups (Fig. 9) were plotted, based on this table. The first column is the table index and lists survey years from 1994 to 2025, annually. The first row contains the column headers corresponding to the six taxonomic groups. A null value indicates that no surveys were conducted or no sightings were recorded for that taxonomic group. The integer values indicate the number of taxa identified to the species level in each year for the corresponding group. File: oo_1789279.csv https://binary.pensoft.net/file/1789279 Jui–Wen Chang

## Figures and Tables

**Table 1.** The sampling methods of nine taxonomic groups in each year. The first row contains the column headers corresponding to the nine taxonomic groups. The first column is the table index and lists only the survey years from 1994 to 2025. A string indicates the sampling methods for that taxonomic group in that year. N/A (Not Applicable / Not Available) could indicate three scenarios: a) There is no survey for that taxonomic group in that year; b) There is a survey for that taxonomic group in that year, yet the record cannot be found; c) The survey scope of these records differs from the specific coverage required for this target taxon in our project.

Year	Avian	Cetacean	Fish	Benthic Invertebrate	Plant	Insect	Spider	Algae	Reptile
1994	Point Count Survey (Mianhua Islet) | Route Survey (Huaping Islet | Sea areas amongst Mianhua, Huaping, and Pengjia Islets)	N/A	N/A	N/A	Line Transect Survey | Quadrat Sampling (10*10 m²)	The survey was conducted without a description of the methods.	N/A	N/A	The survey was conducted without a description of the methods.
1997	N/A	N/A	N/A	N/A	The survey was conducted without a description of the methods.	N/A	N/A	N/A	N/A
2002	Route Survey	N/A	N/A	N/A	N/A	N/A	N/A	N/A	N/A
2003	Route Survey	N/A	N/A	N/A	N/A	N/A	N/A	N/A	N/A
2005	Route Survey (Huaping Islet | Waters off Keelung) | The survey was conducted without a description of the methods. (Mianhua and Pengjia Islets)	N/A	N/A	N/A	The survey was conducted without a description of the methods.	N/A	N/A	N/A	N/A
2008	Route Survey (Huaping Islet | Waters off Keelung) | Line Transect Survey (Mianhua and Pengjia Islets)	N/A	N/A	N/A	Line Transect Survey | Quadrat Sampling (5*5 m²)	Line Transect Survey | Aerial Netting | Host Plant Recognition | Hand Sorting Method | Bait Method	Opportunistic observation during other taxonomic surveys	N/A	The survey was conducted without a description of the methods.
2009	Route Survey (Huaping Islet | Waters off Keelung) | The survey was conducted without a description of the methods. (Mianhua and Pengjia Islets)	N/A	N/A	N/A	The survey was conducted without a description of the methods.	The survey was conducted without a description of the methods.	N/A	N/A	The survey was conducted without a description of the methods.
2010	Route Survey (sea areas | Huaping Islet) | Line Transect Survey (Mianhua Islet, Pengjia Islets) | Point Count Survey (Mianhua Islet) | Area Search Method (Mianhua Islet)	N/A	N/A	N/A	The survey was conducted without a description of the methods.	The survey was conducted without a description of the methods.	N/A	N/A	The survey was conducted without a description of the methods.
2011	Route Survey (sea areas | Huaping Islet) | Line Transect Survey (Mianhua Islet) | Point Count Survey (Mianhua Islet) | Area Search Method (Mianhua, Pengjia Islets)	N/A	Line Transect Survey (30 m * 5 m)	N/A	Line Transect Survey | Quadrat Sampling (Twelve 5*5 m² Quadrats)	Aerial Netting | Sweeping Net Method | Beat Sheet Method | Sieving Method | Aquatic Sieving | Visual Search Method | Trap Method | Bait Method	N/A	N/A	Line Transect Survey
2012	Route Survey (sea areas | Huaping Islet) | Line Transect Survey (Mianhua Islet) | Point Count Survey (Mianhua Islet) | Area Search Method (Mianhua, Pengjia Islets)	N/A	N/A	N/A	Line Transect Survey | Quadrat Sampling (Twelve 5*5 m² Quadrats)	Aerial Netting | Sweeping Net Method | Beat Sheet Method | Sieving Method | Aquatic Sieving | Visual Search Method | Trap Method | Bait Method	N/A	N/A	Line Transect Survey
2013	Route Survey (sea areas | Huaping Islet) | Line Transect Survey (Mianhua Islet) | Point Count Survey (Mianhua Islet) | Area Search Method (Mianhua, Pengjia Islets)	N/A	N/A	N/A	Line Transect Survey | Quadrat Sampling (Twelve 5*5 m² Quadrats)	Aerial Netting | Sweeping Net Method | Beat Sheet Method | Sieving Method | Aquatic Sieving | Visual Search Method | Trap Method | Bait Method	N/A	N/A	Line Transect Survey
2014	Route Survey (sea areas | Huaping Islet) | Line Transect Survey (Mianhua Islet, Pengjia Islets) | Point Count Survey (Mianhua Islet) | Area Search Method (Mianhua Islet)	N/A	N/A	Intertidal Visual Encounter Survey (Crabs)	Line Transect Survey | Quadrat Sampling (Twelve 5*5 m² Quadrats)	Aerial Netting | Sweeping Net Method | Beat Sheet Method | Sieving Method | Aquatic Sieving | Visual Search Method | Trap Method | Bait Method	N/A	N/A	Line Transect Survey
2015	Route Survey (sea areas | Huaping Islet) | Line Transect Survey (Mianhua Islet, Pengjia Islets) | Point Count Survey (Mianhua Islet) | Area Search Method (Mianhua Islet)	N/A	N/A	N/A	Line Transect Survey | Quadrat Sampling (Twelve 5*5 m² Quadrats)	Aerial Netting | Sweeping Net Method | Beat Sheet Method | Sieving Method | Aquatic Sieving | Visual Search Method | Trap Method | Bait Method	N/A	N/A	Line Transect Survey
2016	Route Survey (sea areas | Huaping Islet) | Line Transect Survey (Mianhua Islet, Pengjia Islets) | Area Search Method (Mianhua Islet)	N/A	N/A	N/A	Line Transect Survey | Quadrat Sampling (Twelve 5*5 m² Quadrats)	Aerial Netting | Sweeping Net Method | Beat Sheet Method | Sieving Method | Aquatic Sieving | Visual Search Method | Trap Method | Bait Method	N/A	N/A	Line Transect Survey
2017	Route Survey (sea areas | Huaping Islet) | Line Transect Survey (Mianhua Islet, Pengjia Islets) | Area Search Method (Mianhua Islet)	N/A	N/A	Intertidal Visual Encounter Survey (Crabs)	Line Transect Survey | Quadrat Sampling (Twelve 5*5 m² Quadrats)	Aerial Netting | Sweeping Net Method | Beat Sheet Method | Sieving Method | Aquatic Sieving | Visual Search Method | Trap Method | Bait Method	N/A	N/A	Line Transect Survey
2018	Route Survey (sea areas | Huaping Islet) | Line Transect Survey (Mianhua Islet, Pengjia Islets) | Area Search Method (Mianhua Islet)	N/A	N/A	Intertidal Visual Encounter Survey	Line Transect Survey | Quadrat Sampling (Twelve 5*5 m² Quadrats)	Aerial Netting | Sweeping Net Method | Beat Sheet Method | Sieving Method | Aquatic Sieving | Visual Search Method | Trap Method | Bait Method	N/A	N/A	N/A
2019	Route Survey (sea areas | Huaping Islet) | Line Transect Survey (Mianhua Islet)	Route Survey	N/A	N/A	Line Transect Survey	Visual Search Method | Sweeping Net Method	N/A	N/A	N/A
2020	Route Survey (sea areas | Huaping Islet) | Line Transect Survey (Mianhua, Pengjia Islets) | Area Search Method (Mianhua Islet)	Route Survey	Snorkelling	Snorkelling | Intertidal Visual Encounter Survey	Quadrat Sampling (Twelve 5*5 m² Quadrats)	Aerial Netting | Sweeping Net Method | Beat Sheet Method | Sieving Method | Aquatic Sieving | Visual Search Method | Trap Method | Bait Method	N/A	The survey was conducted without a description of the methods.	Line Transect Survey
2021	Route Survey (sea areas | Huaping Islet) | Area Search Method (Mianhua Islet)	Route Survey	Underwater Visual Census | Roving Diver Method	N/A	Quadrat Sampling (Twelve 5*5 m² Quadrats)	Aerial Netting | Sweeping Net Method | Hand Sorting Method | Sieving Method	N/A	N/A	Opportunistic observation during other taxonomic surveys
2022	Route Survey (sea areas | Huaping Islet) | Area Search Method (Mianhua Islet) | Camera trap (Mianhua Islet)	Route Survey | Line Transect Survey	Underwater Visual Census | Roving Diver Method | Baited Remote Underwater Video | Terrestrial Patrol	Intertidal Line Transect Survey | Intertidal Qualitative Survey | Roving Diver Method	Quadrat Sampling (Twelve 5*5 m² Quadrats) | Quadrat Sampling in a burned area (10*10 m² Quadrat) | Line Transect Survey	Aerial Netting | Sweeping Net Method | Hand Sorting Method | Sieving Method	N/A	N/A	Opportunistic observation during other taxonomic surveys
2023	Route Survey (sea areas | Huaping Islet) | Area Search Method (Mianhua Islet) | Camera trap (Mianhua Islet)	Route Survey	Underwater Visual Census | Roving Diver Method | Baited Remote Underwater Video	Intertidal Qualitative Survey | Roving Diver Method	Quadrat Sampling in a burned area (10*10 m² Quadrat) | Line Transect Survey	Line Transect Survey (Aerial Netting | Sweeping Net Method | Hand Sorting Method | Sieving Method)	N/A	N/A	Opportunistic observation during other taxonomic surveys
2024	Route Survey (sea areas | Huaping Islet) | Area Search Method (Mianhua Islet) | Camera trap (Mianhua Islet)	Route Survey	Underwater Visual Census | Roving Diver Method | Baited Remote Underwater Video | Diver-Operated Video | Intertidal Qualitative Survey	Intertidal Qualitative Survey | Roving Diver Method	N/A	Line Transect Survey (Aerial Netting | Sweeping Net Method | Hand Sorting Method | Sieving Method)	N/A	Intertidal Qualitative Survey	Opportunistic observation during other taxonomic surveys
2025	Route Survey (sea areas | Huaping Islet) | Area Search Method (Mianhua Islet) | Camera trap (Mianhua Islet)	Route Survey	Underwater Visual Census | Baited Remote Underwater Video | Intertidal Qualitative Survey	Intertidal Qualitative Survey | Roving Diver Method | Quadrat Sediment Survey	N/A	Line Transect Survey (Aerial Netting | Sweeping Net Method | Hand Sorting Method | Sieving Method)	N/A	N/A	Opportunistic observation during other taxonomic surveys

**Table 2.** Assignment of "coordinateUncertaintyInMeters" for the nine taxonomic groups, based on survey methodology and spatial scope in the Point-Radius Method.

Taxonomic Groups	Methodology or Area Scope	Coordinate Uncertainty (m)
Avian Species	Precise points of human observation	Estimated offset or 300
	Precise points of machine observation	10
	General observations at Huaping Islet	80
	General observations at Mianhua Islet	400
	General observations at Pengjia Islet	800
	General observations at waters off Keelung	35000
Cetaceans	Cetaceans approached | Distance estimated	≤ 300 or estimated offset
	Maximum visual detection radius (Visual upper bound)	4000
Benthic Invertebrates	Intertidal quadrat	65
	Roving diver method | Qualitative survey	Estimated offset based on survey area
	Unknown historical methods around Mianhua Islet	800
Fish	Precise points of Baited Remote Underwater Video (BRUV)	10
	General observations of BRUV around Mianhua Islet	Expanded to cover multiple survey positions within that year
	Diver-Operated Video (DOV)	100
	Roving diver method | Qualitative survey	Estimated offset
	Underwater Visual Census (UVC) line transect	Estimated offset based on the specific area of the transect lines
	Snorkelling around Mianhua Islet | UVC line transect in 2011	1000
Plants	Specific quadrats (5 x 5 m)	10
	Specific quadrats (10 x 10 m post-fire)	20
	General islet records	400
Insects and Spiders	General islet records	400
Reptiles	Precise points of human or machine observation	10
	General observations of terrestrial species	400
	General observations of marine species	1000 or estimated offset
Algae	Intertidal qualitative survey	70
	Unknown methods around Mianhua Islet	1000

**Table 3.** The central coordinates of thirteen quadrats in the plant survey.

locality	decimalLatitude	decimaLongitude	quadrat (m)	coordinateUncertaintyInMeters
MianhuaIslet_Plant_70	25.48548	122.10439	5 x 5	10
MianhuaIslet_Plant_71	25.48488	122.10485	5 x 5	10
MianhuaIslet_Plant_72	25.48488	122.10495	5 x 5	10
MianhuaIslet_Plant_73	25.48498	122.10509	5 x 5	10
MianhuaIslet_Plant_74	25.48427	122.10484	5 x 5	10
MianhuaIslet_Plant_75	25.48473	122.10604	5 x 5	10
MianhuaIslet_Plant_76	25.4844	122.10626	5 x 5	10
MianhuaIslet_Plant_77	25.48339	122.10557	5 x 5	10
MianhuaIslet_Plant_78	25.48333	122.10418	5 x 5	10
MianhuaIslet_Plant_94	25.48704	122.10489	5 x 5	10
MianhuaIslet_Plant_96	25.48744	122.10523	5 x 5	10
MianhuaIslet_Plant_97	25.48637	122.1061	5 x 5	10
MianhuaIslet_Plant_PostFire	25.48506	122.10434	10 x 10	20
